# *Dclre1c*-Mutation-Induced Immunocompromised Mice Are a Novel Model for Human Xenograft Research

**DOI:** 10.3390/biom14020180

**Published:** 2024-02-02

**Authors:** Yixiao Bin, Sanhua Wei, Ruo Chen, Haowei Zhang, Jing Ren, Peijuan Liu, Zhiqian Xin, Tianjiao Zhang, Haijiao Yang, Ke Wang, Zhuan Feng, Xiuxuan Sun, Zhinan Chen, Hai Zhang

**Affiliations:** 1 Department of Cell Biology, National Translational Science Center for Molecular Medicine, Fourth Military Medical University, Xi’an 710032, China; byx@email.sntcm.edu.cn (Y.B.); novababe@126.com (R.C.); rjing@email.sntcm.edu.cn (J.R.); liupeij0707@aliyun.com (P.L.); xinzhiqian8914@163.com (Z.X.); zhangtianjiao1114@163.com (T.Z.); yhjjqq.love@163.com (H.Y.); wangke@fmmu.edu.cn (K.W.); fengzhuanzhuan@fmmu.edu.cn (Z.F.); sunxiuxuan_709@126.com (X.S.); 2 State Key Laboratory of New Targets Discovery and Drug Development for Major Diseases, Fourth Military Medical University, Xi’an 710032, China; 3 School of Basic Medical Sciences, Shaanxi University of Chinese Medicine, Xianyang 712046, China; 4 Department of Obstetrics and Gynecology, Reproductive Medicine Center, Tang Du Hospital, Fourth Military Medical University, Xi’an 710038, China; sanhuawei3@163.com; 5 Department of Occupational & Environmental Health and the Ministry of Education Key Lab of Hazard Assessment and Control in Special Operational Environment, School of Public Health, Fourth Military Medical University, Xi’an 710032, China; zhanghaowei_syd@aliyun.com

**Keywords:** *dclre1c*, CRISPR/Cas9, knockout, tumor xenograft model, immune reconstitution

## Abstract

Severe combined immunodeficient (SCID) mice serve as a critical model for human xenotransplantation studies, yet they often suffer from low engraftment rates and susceptibility to graft-versus-host disease (GVHD). Moreover, certain SCID strains demonstrate ‘immune leakage’, underscoring the need for novel model development. Here, we introduce an SCID mouse model with a targeted disruption of the *dclre1c* gene, encoding Artemis, which is essential for V(D)J recombination and DNA repair during T cell receptor (TCR) and B cell receptor (BCR) assembly. Artemis deficiency precipitates a profound immunodeficiency syndrome, marked by radiosensitivity and compromised T and B lymphocyte functionality. Utilizing CRISPR/Cas9-mediated gene editing, we generated *dclre1c*-deficient mice with an NOD genetic background. These mice exhibited a radiosensitive SCID phenotype, with pronounced DNA damage and defective thymic, splenic and lymph node development, culminating in reduced T and B lymphocyte populations. Notably, both cell lines and patient-derived tumor xenografts were successfully engrafted into these mice. Furthermore, the human immune system was effectively rebuilt following peripheral blood mononuclear cells (PBMCs) transplantation. The *dclre1c*-knockout NOD mice described herein represent a promising addition to the armamentarium of models for xenotransplantation, offering a valuable platform for advancing human immunobiological research.

## 1. Introduction

The DNA cross-link repair 1C (*dclre1c*) gene, situated on chromosome 2 in mice, orchestrates multiple biological functions through its five transcript variants that give rise to distinct proteins. Notably, transcript variant 201, spanning 16 exons and 15 introns and generating a full-length cDNA of 1461 bp, encodes the Artemis protein with an approximate molecular weight of 88 kDa [1]. The Artemis protein has endonucleolytic activity, preferentially targeting single-stranded DNA and executing 5′-3′ cleavage. This protein is integral in the repair of genotoxic damage, facilitating the non-homologous end joining (NHEJ) pathway of DNA repair; mutations within *dclre1c* can therefore precipitate severe DNA damage [2,3]. Additionally, the Artemis protein is instrumental in V(D)J recombination, a process that is vital for the development and function of T and B lymphocytes. Consequently, mutations in *dclre1c* are associated with severe combined immunodeficiency (SCID), a condition characterized by reduced lymphocyte counts and compromised immune functionality [4,5].

Xenograft models, wherein human tissues or cells are grafted into animal hosts, are pivotal for oncological and immunological research. The choice of laboratory animal is critical to the success of xenografts, with immunodeficient strains being preferred due to their diminished immune cell function and consequent lower rejection rates of foreign tissues than immunocompetent mice [6,7,8]. Commonly utilized strains, such as nude mice, NOD-SCID and NSG mice, offer utility in xenograft applications, but are hampered by poor maternal behaviors and stringent environmental requirements for their upkeep. Some strains also display ‘immune leakiness’, which can confound experimental outcomes [9]. The previous research has demonstrated that the mutation of the *dclre1c* gene in the 129/SvJ inbred mouse strain can lead to a SCID syndrome similar to that in humans, making it a valuable animal model for transplantation studies. However, the *dclre1c*-129/SvJ mutant mice have only been studied for allogeneic hematopoietic stem cell transplantation and no research has been conducted on xenotransplantation applications [10,11,12]. Given the crucial role of Artemis in immune regulation and V(D)J recombination, *dclre1c* mutants have emerged as a potential superior model for xenograft experiments.

The advent of CRISPR/Cas9 gene editing has revolutionized the generation of targeted gene modifications. In this system, a single-guide RNA (sgRNA) directs the Cas9 nuclease to a specific DNA sequence, inducing a double-strand break that, upon cellular repair, results in gene knockout or knockin [13,14]. This study harnesses CRISPR/Cas9 technology to disrupt a segment of exon 10 within the mouse *dclre1c* gene. Our objective is to delineate the resultant mouse phenotype from this gene’s loss of function and to ascertain the suitability of these *dclre1c*-mutant mice as a xenograft model.

## 2. Materials and Methods

### 2.1. Animals

ICR and NOD mice were purchased from Vital River Laboratory Animal Technology Co., Ltd. (Beijing, China) and Gempharmatech Co., Ltd. (Nanjing, China), respectively. The mice were housed and bred in a standard temperature and climate-controlled specific-pathogen-free facility, with a 12 h light/dark cycle and free access to water and food ad libitum. All mouse operations were conducted in compliance with the National Institute of Health’s Guide for the Care and Use of Laboratory Animals, and mouse experiments were approved by the Institutional Animal Care and Use Committee of the National Center for Translational Medicine, Fourth Military Medical University (Approval ID: 2021-NTSCMM-ID014; approval date: 16 December 2021).

### 2.2. Materials and Reagents

Pregnant mare serum gonadotropin (PMSG) and human chorionic gonadotropin (hCG) were purchased from the Ningbo Second Hormone Factory (Ningbo, China). M2, M16 and KSOM embryo culture media were provided by Sigma-Aldrich (St. Louis, MO, USA). Human FITC-CD3, PE-CD4 and APC-CD8 and mouse FITC-CD3, PE-NKp46 and APC-B220 antibodies were purchased from BioLegend (San Diego, CA, USA). Human CD8α (D8A8Y) and CD4 (EP204) rabbit mAb and CD3 mAb for multiplexed immunofluorescence analysis were provided by CST (Danvers, MA, USA) and Proteintech (Rosemont, IL, USA), respectively. RNA extraction kits, cDNA reverse transcriptional kits and mouse genome extraction kits were sourced from TIANGEN Biotech (Beijing, China). DNA damage assay kits using γ-H2AX immunofluorescence were purchased from Beyotime Biotechnology (Shanghai, China). The qPCR kit was provided by Takara Biomedical Technology (Beijing, China). The multiplexed immunofluorescence staining kit was purchased from Akoya Biosciences (Marlborough, MA, USA).

The sgRNA sequence (GCATCAAGCCATCTACCATG) target on exon10 of *declre1c* was ordered and chemically synthesized by Tsingke Biotechnology Co., Ltd. (Beijing, China). Cas9 mRNA was purchased from ThermoFisher Scientific Co., Ltd. (Waltham, MA USA).

Human lung and colon cancer PDX tumor samples (P2) were frozen in liquid nitrogen and kept in our laboratory [15,16].

### 2.3. Microinjection

Female NOD mice (4 w) were used as the embryo donor animals. Superovulation was carried out as previously described [17]. After mating with male NOD mice, fertilized eggs were collected from the ampulla of donor mice. CRISPR/Cas9 method was used to construct *dclre1c* knockout mice. Fertilized eggs were microinjected with 60 mg of *dclre1c* sgRNA and 30 mg of a Cas9 mRNA mixture and cultured at 37 °C for 24 h. The 2-cell forms of the eggs were then transplanted into the ampulla of recipient pseudopregnancy ICR female mice.

### 2.4. Management of the Homozygous Dclre1c Knockout Mouse Colonies

Genome DNA was extracted from the tail tip of 1-week-old F0 mice. The offspring genotypes were identified via PCR with forward and reverse primers (Table 1). After purification, PCR products were sequenced with Sanger sequencing and the results were analyzed with SnapGene 6.0.2 software (GSL Biotech, Chicago, IL, USA).

PCR-confirmed founder mice were mated with wildtype NOD mice to generate F1; positive F1 animals derived from same genotype founder mice were intercrossed to produce F2. Heterozygous F2 animals were intercrossed until obtaining homozygous mice (*dclre1c*-NOD mice).

### 2.5. Organ Index and Hematoxylin and Eosin Staining (H&E) Assay

*dclre1c*-NOD mice were euthanized, and the organs of interest (thymus, spleen and inguinal, axillar, cervical, mesenteric lymph nodes) were collected for histological examination and organ index calculation. Spleen and thymus tissues were washed with sterile water, dried with filter paper, and weighed accurately to calculate the organ index. The following formula was used for this calculation: organ index (mg/g) = organ weight (mg)/body weight (g).

Samples of spleen, thymus and lymph nodes were fixed in 4% paraformaldehyde. After embedding, the tissue was cut into thin slices and stained according to the instructions of the hematoxylin and eosin staining kit. The stained sections were observed and photographed via a microscope.

### 2.6. Flow Cytometry

An amount of 50 μL of peripheral blood was collected in preservative-free heparin from the tail veins of *dclre1c*-NOD mice. Samples were lysed with erythrocyte lysing solution and labeled with 1:1000-diluted FITC-CD3, PE-NKp46 and APC-B220 antibodies for 30 min at room temperature in a dark place. Then, samples were analyzed using BD LSRFortessa flow cytometry (Becton, Dickinson and Company, Franklin Lakes, NJ, USA) and data were analyzed using FlowJo software V10.6.2 (FlowJo LLC, Ashland, OR, USA).

### 2.7. Cell Culture

Human lung adenocarcinoma A549 and T lymphobla stoid Jurkat cell lines, carrying the luciferase reporter gene, were purchased from the Type Culture Collection of the Chinese Academy of Sciences (Shanghai, China). Cells were cultured in high-glucose Dulbecco’s modified eagle medium or Roswell Park Memorial Institute 1640 supplemented with 10% fetal bovine serum and 100 μL/mL penicillin/streptomycin under a humidified atmosphere of 5% CO_2_ at 37 °C.

### 2.8. Real Time qPCR

Total RNA was extracted from the hearts, livers, spleens, brains, lungs and kidneys of *dclre1c*-NOD and wildtype NOD mice using a total RNA extraction kit following the manufacturer’s instructions. For cDNA synthesis, a 20 μL reaction system containing 100 ng of extracted RNA and 10 pM oligo(dT)18 primers was prepared. For real-time qPCR, 2 μL cDNA, 12.5 μL of TB Green Premix Ex Taq II, 1 μL of forward and reverse primers and 8.5 μL of nuclease-free H_2_O were added to a 25 μL reaction system. Amplification was performed using a real-time PCR (RT-qPCR) system. RT-qPCR results were analyzed using the expression of glyceraldehyde-3-phosphate dehydrogenase (GAPDH) as the internal reference; The relative mRNA expression was quantified as 2^−∆∆Ct^ as described previously [18], in which Ct is the threshold cycle number and where ΔCT was calculated by the Ct value of internal control GADPH from the Ct value of genes. The primer sequences used are listed in Table 1.

### 2.9. DNA Damage Assay

*dclre1c*-NOD and wildtype NOD mice were irradiated with 2.5 Gy ^60^Co, and 4 h later, mice were sacrificed. Hearts, livers, spleens, brains, lungs and kidneys were resected and placed in a cryomold with an optimal cutting temperature (OCT) compound. The cryomold was placed in liquid nitrogen until the OCT compound froze. The frozen sections were cut to 5 μm thickness. After washing with phosphate-buffered saline (PBS, pH 7.4) and drying at room temperature for 2 h, sections were analyzed via with γ-H2AX immunofluorescence for DNA damage analysis according to the manufacturer’s instructions. Briefly, sections were fixed, blocked and then incubated with primary anti-γ-H2AX Rabbit mAb overnight at 4 °C. After washing, secondary Alexa Fluor 488 anti-rabbit antibody was added and the nucleus was stained with DAPI; sections were observed under a Leica STELLARIS 5WLL confocal microscope (Leica Microsystems, Wetzlar, Germany).

### 2.10. Human Xenograft Model

*dclre1c*-NOD and wildtype NOD mice (6 w) were injected intravenously with 5 × 10^6^ A549- or Jurkat-Luc cells to construct a human tumor xenograft model; mice were imaged using an IVIS Lumina II imaging system (PerkinElmer, Waltham, MA, USA) at different time point after inoculation. A human PBMC transplanted model was established as previously reported [19]. Briefly, PBMCs were isolated and purified via Ficoll density centrifugation. After counting, 5 × 10^6^ PBMCs were inoculated intravenously into 2.5 Gy ^60^Co-irradiated or non-irradiated *dclre1c*-NOD and wildtype NOD mice. Three weeks later, 50 μL of peripheral blood was collected and stained with human FITC-CD3, PE-CD4 and APC-CD8 antibodies; samples were analyzed via flow cytometry.

### 2.11. Multiplexed Immunofluorescence

Immunofluorescence assays for human CD3+, CD4+ and CD8+ T-cell subtypes were performed with tissue sections of PBMC-transplanted mice according to the manufacture’s protocol. Briefly, after being deparaffinized and dehydrated, slides of the thymus, spleen and lymph nodes were immersed in Tris-EDTA antigen retrieval buffer and subjected to heat-induced antigen retrieval. To block endogenous peroxidases and antigens, 3% H_2_O_2_ and 3% bovine serum albumin (BSA) were used, respectively. Slides were then sequentially incubated with anti-CD3 (1:250), anti-CD4 (1:50) and anti-CD8α (1:100) antibodies. After washing, nuclei were counterstained with DAPI. Slides were then scanned using a Vectra Polaris Automated Quantitative Pathology Imaging System (Marlborough, MA, USA).

### 2.12. Statistical Analysis

Excel 2019 and GraphPad Prism 8.0.2 (GraphPad, San Diego, CA, USA) were used for statistical analyses. A Student’s *t* test and one-way analysis of variance were performed to compare the significant differences between wildtype and *dclre1c*^−/−^ mice. The values are expressed as means ± SD in bar graphs, and values with a *p*-value less than 0.05 are considered significant. Statistical significance is represented as *** (*p*-value ≤ 0.001), ** (*p*-value ≤ 0.01), * (*p*-value ≤ 0.05).

## 3. Results

### 3.1. Generation of Homozygous Dclre1c Knockout Mouse Colonies

**Figure 1:**
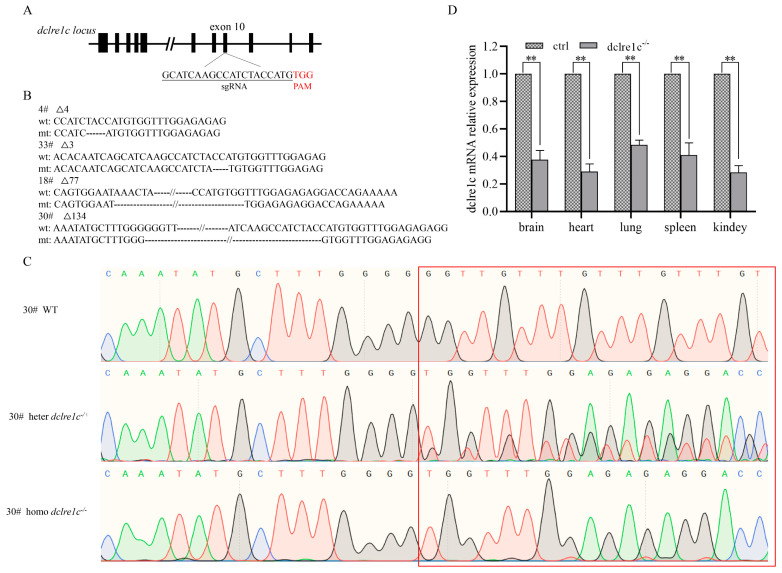
*dclre1c*−knockout mouse is constructed with CRISPR/Cas9 technology. Schema and sequence of sgRNAs targeted on *dclre1c* exon10 locus (**A**). Genotype sequence of wildtype and mutated mice in target region (**B**), # indicates the number of mutation mice. Sanger sequencing maps of wildtype, heterozygotes and homozygotes of *dclre1c*−NOD mice, red box indicates the comparison of wild-type, heterozygous, and homozygous genotypes in target region. (**C**). *dclre1c* mRNA relative expression in different organs of knockout mice (**D**). Data are means ± SD of three independent experiments performed in triplicate (** *p* ≤ 0.01, compared with wildtype).

The *dclre1c* gene, known to encode the Artemis protein, is implicated in immune system function and the repair of double-strand breaks (DSBs) during V(D)J recombination. Utilizing CRISPR/Cas9-mediated gene editing, we sought to create a new strain of SCID mice with a targeted knockout of *dclre1c*. We designed specific sgRNAs against exon 10 of *dclre1c*, and the resulting sgRNA-Cas9 mRNA complex was microinjected into the cytoplasm of fertilized oocytes (Figure 1A). Following a gestation period of 21 days, the resulting offspring were genotyped using PCR and Sanger sequencing. Of the thirty-three pups born, six founders carried four distinct genotypes, each characterized by deletions of varying lengths in the targeted region (Figure 1B). The Δ134 bp mutation, which encompassed a substantial portion of exon 10 and the adjacent intronic sequence, was predominant. Consequently, founder mouse #30, harboring this deletion, was chosen for further breeding with wild-type NOD mice. This cross produced heterozygous progenies, which were subsequently interbred to yield homozygous *dclre1c*-NOD offspring (Figure 1C). Analysis of *dclre1c* mRNA expression in various organs of the *dclre1c*-NOD mice revealed a significant reduction in transcriptional levels (Figure 1D), confirming the successful knockout of exon 10. However, due to the complexity of the gene and the issue of primers position, *dclre1c* knockout did not have a very pronounced effect on transcriptional levels, relative *dclre1c* mRNA expression maintained almost 50% in some organ, like lung, spleen. Collectively, these results demonstrate the successful generation of *dclre1c* exon 10 knockout mice, providing a novel mouse model for further study.

### 3.2. Dclre1c Knockout Aggravates DNA Damage

**Figure 2:**
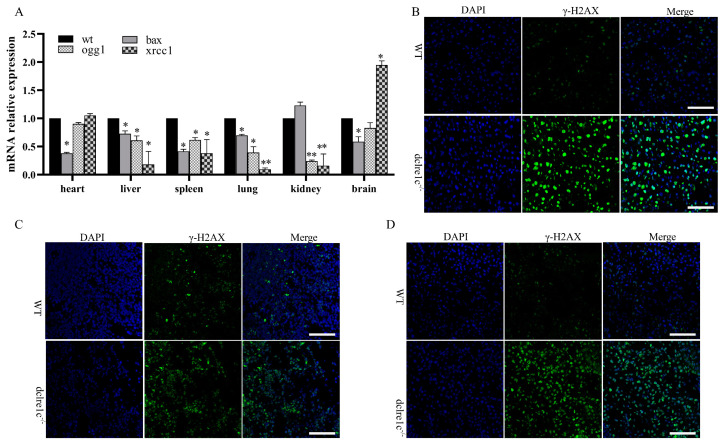
DNA damage is aggravated in *dclre1c*-NOD mice. mRNA expression of DNA-repair-related genes in wildtype NOD and *dclre1c*−NOD mice (**A**). γ−H2AX expression detection with immunofluorescent in the liver, spleen and kidneys of wildtype NOD and *dclre1c*−NOD mice (**B**–**D**). Scar bar = 60 μm. Data are means ± SD of three independent experiments performed in triplicate (* *p* ≤ 0.05, ** *p* ≤ 0.01, compared with wildtype).

The Artemis protein, encoded by the *dclre1c* gene, is essential for DNA repair, possessing single-strand-specific 5′-3′ exonuclease activity. Loss of function in this gene abrogates its endonucleolytic activity, crucial for resolving 5′ and 3′ hairpins and overhangs, thus exacerbating DNA damage. Exposure of mice to 2.5 Gy of ^60^Co radiation resulted in diminished mRNA expressions of DNA repair genes, including *bax* and *ogg1*, in *dclre1c*-NOD mice, indicating an intensification of DNA damage due to the mutation (Figure 2A). Conversely, an increase in *xrcc1* expression was observed in the heart and more prominently in the brain, suggesting a milder extent of DNA damage in these organs (Figure 2A). These molecular changes were substantiated through immunofluorescence staining for the DNA damage marker γ-H2AX. A significant elevation in γ-H2AX expression was noted in the liver, spleen, and kidneys of *dclre1c*-NOD mice (Figure 2B–D). However, changes in γ-H2AX expression in the brain, heart and lungs were not notable. Collectively, these findings corroborate previous reports and validate the *dclre1c*-NOD mouse model as one with exacerbated DNA damage upon irradiation [20].

### 3.3. Dclre1c Knockout Obstructs Immune Organ Development

**Figure 3:**
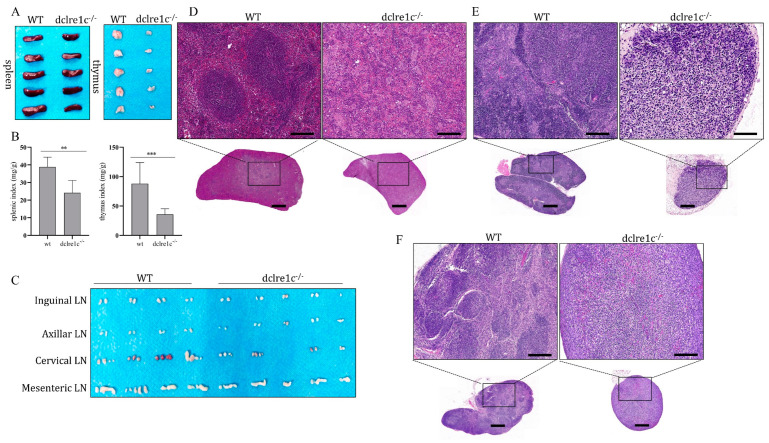
*dclre1c* knockout destructs immune organ development. Gross anatomical morphology of the thymus and spleen in wildtype NOD and *dclre1c*−NOD mice (**A**). Various organs were extracted and the organ/weight ratios were calculated (**B**). Gross anatomical morphology of different lymph nodes in wildtype NOD and *dclre1c*−NOD mice (**C**). Spleen, thymus and lymph node tissues were sectioned and stained via the H&E method (**D**–**F**). Scar bar = 1 mm for panorama tissue sections. Magnification scar bar = 20 μm. Data are means ± SD of three independent experiments performed in triplicate (** *p* ≤ 0.01, *** *p* ≤ 0.001, compared with wildtype).

The *dclre1c* gene plays a pivotal role in V(D)J recombination, and its disruption can result in developmental anomalies within the immune system. To evaluate the impact of *dclre1c* deficiency of lymphoid organogenesis, we assessed the development of central and peripheral lymphoid organs in *dclre1c*-NOD mice. Our observations revealed a marked reduction in the size of the thymus and spleen, as evidenced by decreased organ indices (Figure 3A,B). Notably, thymic development was severely compromised, with *dclre1c*-NOD mice exhibiting a substantial reduction in thymus size compared to wildtype controls. Furthermore, lymph node (LN) formation was affected both in terms of number and size, particularly within the cervical and mesenteric regions (Figure 3C). Structural examination revealed considerable reductions in the volume of the thymus, spleen and cervical LNs in the mutant mice, accompanied by profound architectural disruption. The spleen’s white pulp was notably absent, replaced by the proliferation of megakaryocytes and myeloid progenitors within the sinuses (Figure 3D). In the thymus, the cortex and medulla were significantly reduced, with blurred corticomedullary demarcation and a sparse presence of lymphoblastic precursors (Figure 3E). The cervical lymph nodes exhibited losses of lymphoid follicles and mature plasma cells, highlighting the extensive structural aberrations (Figure 3F).

### 3.4. Dclre1c Knockout Contributes to Immune Cell Insufficiecy

**Figure 4:**
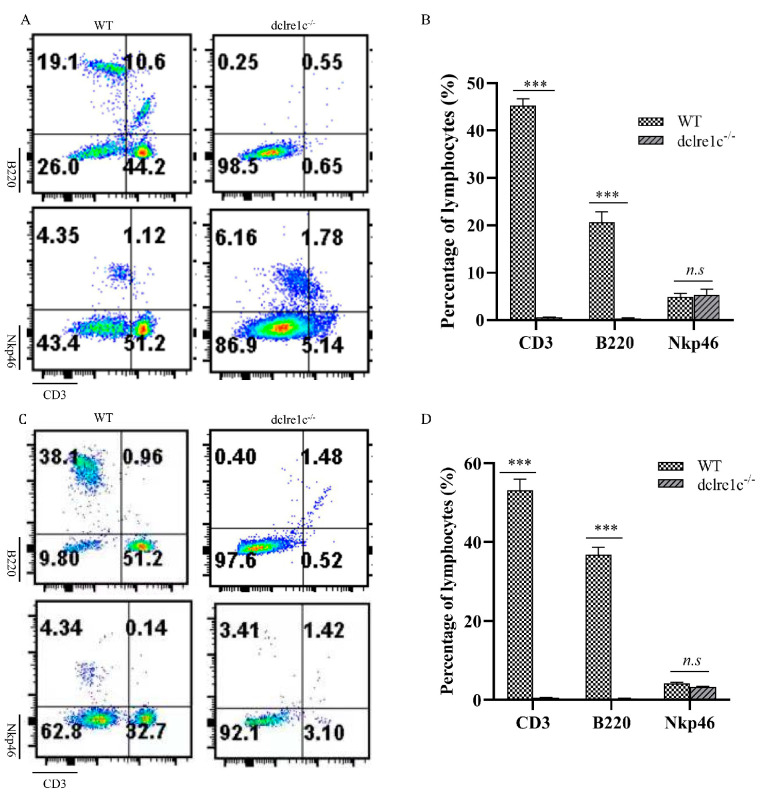
*dclre1c* knockout decreases the percentage of immune cells. Flow cytometric analyses of immune cells in peripheral blood (**A**,**B**). Representative dot−plots (**A**) and quantitative assessment of the percentage of CD3+, B220+ and Nkp46+ cells ((**B**), pooled from three independent experiments) are shown. Flow cytometric analyses of immune cells in spleen (**C**,**D**). Representative dot−plots (**C**) and quantitative assessment of the percentage of CD3+, B220+ and Nkp46+ cells ((**D**), pooled from three independent experiments) are shown. Data are means ± SD of three independent experiments performed in triplicate (*** *p* ≤ 0.001, compared with wildtype. *n.s*: no significance).

The removal of *dclre1c* prompted us to investigate its impact on lymphoid organogenesis and consequent lymphocyte lineage cell development. To this end, we analyzed T, B and NK cell populations in the peripheral blood and spleens of *dclre1c*-NOD mice using flow cytometry. Our findings revealed a significant reduction in CD3+ T cells and B220+ B cells in both peripheral blood (Figure 4A,B) and the spleen (Figure 4C,D). Conversely, NK cell population remained unaffected by the loss of *dclre1c*, indicating the gene’s selective importance in the ontogeny and differentiation of T and B lymphocytes, rather than NK cells.

### 3.5. Immunodeficiency Caused by Dclre1c Knockout Is Suitable for Human Tumor Xenograft Models

**Figure 5:**
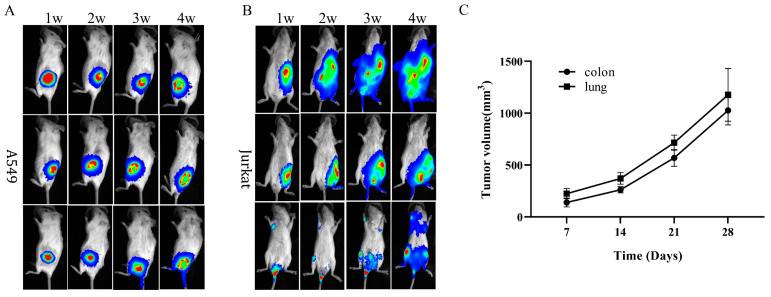
A human tumor xenograft model is established in *dclre1c*-NOD mice with cell-line-derived and patient-derived samples. Luciferase bioluminescence imaging of cell-line-derived xenograft model after incubation with A549 and Jurkat cells (**A**,**B**). Growth curve of human lung and colon patient-derived xenografts (**C**).

The developmental aberrations observed in T and B cell lineages affirm the classification of the *dclre1c* knockout mouse as a novel SCID phenotype. Preceding studies have established the utility of SCID mouse models, such as NOD-SCID, C.B17-SCID and SCID beige, in human xenograft research [21,22,23]. In this vein, we explored the aptitude of *dclre1c*-knockout-mediated SCID mice to serve as hosts for human xenografts. Following inoculation with A549 lung adenocarcinoma and Jurkat T lymphoblastoid cells, subcutaneous and systemic metastatic models were successfully established over time (Figure 5A,B).

Patient-derived xenografts (PDXs), which involve the transplantation of clinical tumor tissues into immunodeficient mice, represent a more complex model than those derived from cell lines. In our study, lung and colon cancer PDX tumor samples (P2) were successfully engrafted subcutaneously into *dclre1c*-NOD mice. Within seven days post-transplantation, palpable subcutaneous tumors were detected, and these tumors exhibited progressive growth (Figure 5C). This evidence supports the suitability of *dclre1c*-NOD mice as a robust model for PDX construction, thus highlighting their potential in human tumor xenograft research.

### 3.6. Dclre1c Knockout Mice Are Ideal for Human Immune System Reconstitution

**Figure 6:**
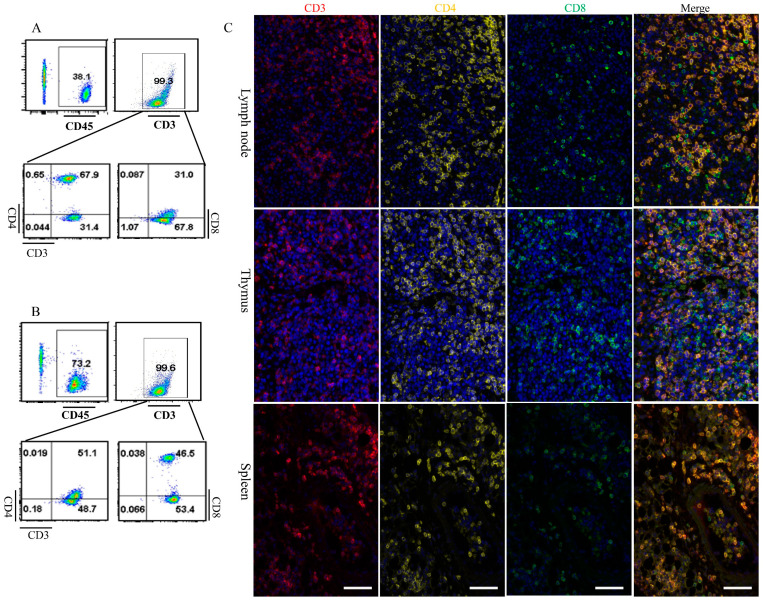
Human immune system reconstitution in *dclre1c*-NOD mice. Flow cytometric analyses of immune cells in peripheral blood (**A**). Representative dot-plots are shown and numbers indicate proportion of human CD45+ (upper left), CD3+ (upper right), CD4+ (lower left) and CD8+ (lower right) amongst of CD3+ cells. Flow cytometric analyses of immune cells in spleen (**B**). Representative dot-plots are shown and numbers indicate proportion of human CD45+ (upper left), CD3+ (upper right), CD4+ (lower left) and CD8+ (lower right) amongst of CD3+ cells. Human CD3+, CD4+ and CD8+ T cell detected via multiplexed immunofluorescence ((**C**), red CD3+, yellow CD4+, green CD8+ T cell). Scar bar = 60 μm.

SCID mice are instrumental in reconstituting the human immune system [24,25]. We explored this potential using *dclre1c*-NOD mice, assessing their suitability for human immune system reconstitution. Human peripheral blood mononuclear cells (PBMCs) were intravenously administered into both ^60^Co-irradiated and non-irradiated *dclre1c*-NOD mice. Three weeks post-transplantation, human CD45+ T cells were exclusively detected in the peripheral blood and splenic cell suspensions of the irradiated cohort. Notably, CD3+CD4+ helper and CD3+CD8+ cytotoxic T cells constituted the majority of the CD45+ T cell population, exhibiting both proliferation in peripheral blood (Figure 6A) and chimerism within splenic tissues (Figure 6B). However, other lymphoid cells, such as B cells, NK cells, and myeloid cells could not be detected. Multiplex immunofluorescence further confirmed that human CD3+, CD4+ and CD8+ T cells underwent chimeric proliferation in the thymus, lymph nodes and spleens of the irradiated *dclre1c*^−/−^ mice (Figure 6C). Unfortunately, human immune system could not be successfully reconstituted in wildtype NOD mice. These findings collectively suggest that the *dclre1c*-NOD mouse model represents a novel and promising platform for human immune system reconstitution and xenograft research.

## 4. Discussion

The V(D)J recombination mechanism is fundamental to the assembly of T cell receptors (TCRs) and B cell receptors (BCRs) [26], essential processes for adaptive immunity. Dysregulation of V(D)J recombination has been implicated in the onset of SCID and an increased susceptibility to radiation-induced cellular damage [27,28]. The *dclre1c* gene, a component of the non-homologous end-joining (NHEJ) pathway, is pivotal for DNA repair and V(D)J recombination. Mutations in this gene have been established as a causative factor for SCID [29]. Our research has successfully generated a *dclre1c* mutated immunodeficient rodent model based on NOD mice, which had similar biological characteristics with NOD-SCID mice. *dclre1c*-NOD mouse not only contained T, B cells severe combined immunodeficiency, but maintained biological characteristics of NOD mice, such as low function of NK cells, absent of complement and macrophages [30]. Theoretically, *dclre1c* mutation in NOD mice was more immunocompromised than 129/SvJ mutation mice, but not as much as *prkdc* and *IL2rg* double genes mutated NSG or NOG mice. The findings from this study not only confirm the development of characteristic SCID symptoms, but also reveal an enhanced radiosensitivity in the mutant mice, highlighting their utility in modeling human xenografts.

Genetic aberrations in several NHEJ pathway constituents, such as *prkdc*, *dclre1c*, *rag1/rag2*, *Ku70/80*, *XRCC4* and *Ligase IV*, have been associated with the manifestation of SCID, typified by the concurrent deficiency of T and B cells [31,32,33]. Our research corroborates previous findings, where both heterozygous and homozygous deletions, along with missense and point mutations at the exon or splice donor sites of *dclre1c*, contribute to radiosensitive SCID (RS-SCID) [3,5,34]. Specifically, we have demonstrated that a 134 bp deletion within exon 10 of *dclre1c* precipitates SCID, characterized by the absence of T and B cells in the mice. The *dclre1c* gene encodes the Artemis protein, which in conjunction with PRKDC forms a complex integral to the NHEJ repair mechanism [35]. Upon initiation of V(D)J recombination and detection of DNA double-strand breaks (DSBs), the Artemis–PRKDC complex orchestrates the repair of DSBs, thus enabling the proper assembly of TCRs and BCRs and maintaining their diversity [36,37]. Mutations in *dclre1c* disrupt the formation of the Artemis–PRKDC complex, leading to defective NHEJ, failure of V(D)J recombination and, ultimately, the development of SCID.

The fidelity of TCR and BCR assembly is ensured by precise V(D)J rearrangement, which is mediated by a cohort of genes. Mutations within these genes can lead to a spectrum of immunodeficiency phenotypes. The *rag1/rag2* complex initiates this process by recognizing and binding to recombination signal sequences (RSSs) adjacent to the V and J gene segments, facilitating the formation of a synaptic complex [38]. After RSS cleavage, the DNA ends are bound by the *Ku70/80* heterodimer, which recruits the catalytic subunit of DNA-dependent protein kinases (DNA-PKcs) to activate the NHEJ machinery [39]. DNA-PKcs phosphorylates Artemis, enabling the Artemis–DNA-PKcs complex to open hairpin-sealed coding ends. This prepares the DNA for ligation by Ligase IV, culminating in the completion of V(D)J rearrangement [40]. *rag1/rag2* plays a role in the initiation of V(D)J rearrangement. Its mutations completely abrogate the development of T and B cells, causing typical SCID without immune leakiness [41]; however, mutations in gene-encoding components of the NHEJ pathway, such as *prkdc*, *Ku70/80* and *Ligase IV*, result in a radiosensitive form of SCID with immune leakiness [42]. Our observations of *dclre1c* mutant mice, which do not include the increase in T cell populations or immunoglobulin expression typically associated with immune leakage, suggest a unique phenotype distinct from *prkdc*-related defects. This discrepancy could be due to differences in genetic backgrounds, as indicated by contrasting results from Artemis-deficient 129/SvJ mice [43].

In xenograft transplantation research, the level of host immunodeficiency is correlated with the success rate of graft acceptance. Our *dclre1c* mutants demonstrated a lower rejection of xenografts, including human-tumor-derived cell lines, primary tumor tissues and peripheral blood mononuclear cells (PBMCs), with proliferative human T cell subsets observable post-transplantation. Although a direct comparison with other immunodeficient mouse models was not conducted, these findings suggest the potential utility of *dclre1c* mutants in xenograft studies. We also noted that human PBMCs proliferated exclusively in irradiated *dclre1c* mutants, indicating their radiosensitivity—a trait potentially advantageous for certain experimental paradigms—and also highlighting an area for refinement in comparison to NSG and BRG mice. Building upon the success of these models, future efforts will focus on introducing mutations into the *IL2rg* gene in *dclre1c* mutants. The aim of this is to attenuate responses mediated by NK cells and cytokines such as IL-2, IL-4, IL-7, IL-9, IL-15 and IL-21, thereby enhancing the engraftment efficiency for xenograft modeling [17,44,45].

## Figures and Tables

**Table 1:** Primer sets used for sequence and qPCR.

Name	Forward Primer	Reverse Primer
Dclre1c-seq	AAAACCTCATCTGCAATTGCTTTTA	CTTGGGCTTGTGCTGATGTG
Dclre1c-Qpcr	TGAGGCTTCGGGTGAGAAGG	AACTGATCCTGGGCAGTGAC
Bax	AAACTGGTGCTCAAGGCCC	GGTCCCGAAGTAGGAGAGGA
Xrcc1	TCCGTCCGTCTGTTTGTCTG	GCTTCCTGGGAACCTGTTGT
Ogg1	GAGACGACAGCCAGGCCTTT	GGAGGTTTGGGAAGCCATGAT
GAPDH	ACCCTTAAGAGGGATGCTGC	CCCAATACGGCCAAATCCGT

## Data Availability

The data presented in this study are available in this article.

## References

[1] Rooney S., Sekiguchi J., Zhu C., Cheng H.-L., Manis J., Whitlow S., DeVido J., Foy D., Chaudhuri J., Lombard D. (2002). Leaky Scid Phenotype Associated with Defective V(D)J Coding End Processing in Artemis-Deficient Mice. Mol. Cell.

[2] Watanabe G., Lieber M.R. (2022). Dynamics of the Artemis and DNA-PKcs Complex in the Repair of Double-Strand Breaks. J. Mol. Biol..

[3] Watanabe G., Lieber M.R., Williams D.R. (2022). Structural Analysis of the Basal State of the Artemis: DNA-PKcs Complex. Nucleic Acids Res..

[4] Strubbe S., De Bruyne M., Pannicke U., Beyls E., Vandekerckhove B., Leclercq G., De Baere E., Bordon V., Vral A., Schwarz K. (2021). A Novel Non-Coding Variant in DCLRE1C Results in Deregulated Splicing and Induces SCID through the Generation of a Truncated ARTEMIS Protein That Fails to Support V(D)J Recombination and DNA Damage Repair. Front. Immunol..

[5] Moshous D., Callebaut I., de Chasseval R., Corneo B., Cavazzana-Calvo M., Le Deist F., Tezcan I., Sanal O., Bertrand Y., Philippe N. (2001). Artemis, a novel DNA Double-strand Break Repair/V(D)J Recombination Protein, is Mutated in Human Severe Combined Immune Deficiency. Cell.

[6] Wang W., Li Y., Lin K., Wang X., Tu Y., Zhuo Z. (2023). Progress in Building Clinically Relevant Patient-derived Tumor Xenograft Models for Cancer Research. Anim. Model. Exp. Med..

[7] Tracey A.T., Murray K.S., Coleman J.A., Kim K. (2020). Patient-Derived Xenograft Models in Urological Malignancies: Urothelial Cell Carcinoma and Renal Cell Carcinoma. Cancers.

[8] Shultz L.D., Goodwin N., Ishikawa F., Hosur V., Lyons B.L., Greiner D.L. (2014). Human Cancer Growth and Therapy in Immunodeficient Mouse Models. Cold Spring Harb. Protoc..

[9] Tanaka T., Nishie R., Ueda S., Miyamoto S., Hashida S., Konishi H., Terada S., Kogata Y., Sasaki H., Tsunetoh S. (2021). Patient-Derived Xenograft Models in Cervical Cancer: A Systematic Review. Int. J. Mol. Sci..

[10] Benjelloun F., Garrigue A., Chappedelaine C.D.-D., Soulas-Sprauel P., Malassis-Séris M., Stockholm D., Hauer J., Blondeau J., Rivière J., Lim A. (2008). Stable and functional lymphoid reconstitution in artemis-deficient mice following lentiviral artemis gene transfer into hematopoietic stem cells. Mol. Ther..

[11] Xiao Z., Dunn E., Singh K., Khan I.S., Yannone S.M., Cowan M.J. (2009). A non-leaky Artemis-deficient mouse that accurately models the human severe combined immune deficiency phenotype, including resistance to hematopoietic stem cell transplantation. Biol. Blood Marrow Transpl..

[12] Li L., Salido E., Zhou Y., Bhattacharyya S., Yannone S.M., Dunn E., Meneses J., Feeney A.J., Cowan M.J. (2005). Targeted disruption of the Artemis murine counterpart results in SCID and defective V(D)J recombination that is partially corrected with bone marrow transplantation. J. Immunol..

[13] Bhatia S., Pooja, Yadav S.K. (2023). CRISPR-Cas for Genome Editing: Classification, Mechanism, Designing and Applications. Int. J. Biol. Macromol..

[14] Yang Y., Huang Y. (2019). The CRIPSR/Cas Gene-editing System—An Immature but Useful Toolkit for Experimental and Clinical Medicine. Anim. Model. Exp. Med..

[15] Chen X.-H., Chen R., Shi M.-Y., Tian R.-F., Zhang H., Xin Z.-Q., Chen Z.-N., Wang K. (2022). Chimeric Antigen Receptor T Cells Targeting CD147 for Non-small Cell Lung Cancer Therapy. Transl. Oncol..

[16] Cui H., Wei W., Qian M., Tian R., Fu X., Li H., Nan G., Yang T., Lin P., Chen X. (2022). PDGFA-associated Protein 1 is a Novel Target of C-Myc and Contributes to Colorectal Cancer Initiation and Progression. Cancer Commun..

[17] Zhao Y., Liu P., Xin Z., Shi C., Bai Y., Sun X., Zhao Y., Wang X., Liu L., Zhao X. (2019). Biological Characteristics of Severe Combined Immunodeficient Mice Produced by CRISPR/Cas9-Mediated Rag2 and IL2rg Mutation. Front. Genet..

[18] Schmittgen T.D., Livak K.J. (2008). Analyzing real-time PCR data by the comparative C(T) method. Nat. Protoc..

[19] Yue X., Petersen F., Shu Y., Kasper B., Magatsin J., Ahmadi M., Yin J., Wax J., Wang X., Heidecke H. (2021). Transfer of PBMC From SSc Patients Induces Autoantibodies and Systemic Inflammation in Rag2-/-/IL2rg-/- Mice. Front. Immunol..

[20] Bétous R., de Rugy T.G., Pelegrini A.L., Queille S., de Villartay J.-P., Hoffmann J.-S. (2018). DNA Replication Stress Triggers Rapid DNA Replication Fork Breakage by Artemis and XPF. PLOS Genet..

[21] Gill R.P.K., Gantchev J., Villarreal A.M., Ramchatesingh B., Netchiporouk E., Akilov O.E., Ødum N., Gniadecki R., Koralov S.B., Litvinov I.V. (2022). Understanding Cell Lines, Patient-Derived Xenograft and Genetically Engineered Mouse Models Used to Study Cutaneous T-Cell Lymphoma. Cells.

[22] Eiseman J., Lan J., Guo J., Joseph E., Vucenik I. (2011). Pharmacokinetics and Tissue Distribution of Inositol Hexaphosphate in C.B17 SCID Mice Bearing Human Breast Cancer Xenografts. Metabolism.

[23] Villodre E.S., Hu X., Eckhardt B.L., Larson R., Huo L., Yoon E.C., Gong Y., Song J., Liu S., Ueno N.T. (2022). NDRG1 in Aggressive Breast Cancer Progression and Brain Metastasis. JNCI J. Natl. Cancer Inst..

[24] Stripecke R., Münz C., Schuringa J.J., Bissig K., Soper B., Meeham T., Yao L., Di Santo J.P., Brehm M., Rodriguez E. (2020). Innovations, Challenges, and Minimal Information for Standardization of Humanized Mice. EMBO Mol. Med..

[25] De Barros S.C., Zimmermann V.S., Taylor N. (2013). Concise Review: Hematopoietic Stem Cell Transplantation: Targeting the Thymus. Stem Cells.

[26] Muljo S.A., Schlissel M.S. (2000). Pre-B and Pre-T-cell Receptors: Conservation of Strategies in Regulating Early Lymphocyte Development. Immunol. Rev..

[27] Lee Y.-R., Kang G.-S., Oh T., Jo H.-J., Park H.-J., Ahn G.-O. (2023). DNA-Dependent Protein Kinase Catalytic Subunit (DNA-PKcs): Beyond the DNA Double-Strand Break Repair. Mol. Cells.

[28] Rivera-Munoz P., Abramowski V., Jacquot S., André P., Charrier S., Lipson-Ruffert K., Fischer A., Galy A., Cavazzana M., de Villartay J.-P. (2016). Lymphopoiesis in Transgenic Mice Over-expressing Artemis. Gene Ther..

[29] Lee P.P., Woodbine L., Gilmour K.C., Bibi S., Cale C.M., Amrolia P.J., Veys P.A., Davies E.G., Jeggo P.A., Jones A. (2013). The Many Faces of Artemis-deficient Combined Immunodeficiency-Two Patients with DCLRE1C Mutations and a Systematic Literature Review of Genotype-phenotype Correlation. Clin. Immunol..

[30] Chen J., Liao S., Xiao Z., Pan Q., Wang X., Shen K., Wang S., Yang L., Guo F., Liu H.-F. (2022). The development and improvement of immunodeficient mice and humanized immune system mouse models. Front. Immunol..

[31] Jiang W., Estes V.M., Wang X.S., Shao Z., Lee B.J., Lin X., Crowe J.L., Zha S. (2019). Phosphorylation at S2053 in Murine (S2056 in Human) DNA-PKcs Is Dispensable for Lymphocyte Development and Class Switch Recombination. J. Immunol..

[32] Bañuelos C., Banáth J., MacPhail S., Zhao J., Eaves C., O’connor M.D., Lansdorp P., Olive P. (2008). Mouse but not Human Embryonic Stem Cells are Deficient in Rejoining of Ionizing Radiation-induced DNA Double-strand Breaks. DNA Repair.

[33] Roch B., Abramowski V., Etienne O., Musilli S., David P., Charbonnier J.B., Callebaut I., Boussin F.D., de Villartay J.P. (2021). An XRCC4 Mutant Mouse, a Model for Human X4 Syndrome, Reveals Interplays with Xlf, PAXX, and ATM in Lymphoid Development. eLife.

[34] Morio T. (2017). Recent Advances in the Study of Immunodeficiency and DNA Damage Response. Int. J. Hematol..

[35] Kumrah R., Vignesh P., Patra P., Singh A., Anjani G., Saini P., Sharma M., Kaur A., Rawat A. (2019). Genetics of Severe Combined Immunodeficiency. Genes Dis..

[36] Akamatsu Y., Oettinger M.A. (1998). Distinct Roles of RAG1 and RAG2 in Binding the V(D)J Recombination Signal Sequences. Mol. Cell. Biol..

[37] Allam A., Kabelitz D. (2006). TCR trans-Rearrangements: Biological Significance in Antigen Recognition vs the Role as Lymphoma Biomarker. J. Immunol..

[38] Bassing C.H., Swat W., Alt F.W. (2002). The Mechanism and Regulation of Chromosomal V(D)J Recombination. Cell.

[39] Chen X., Xu X., Chen Y., Cheung J.C., Wang H., Jiang J., de Val N., Fox T., Gellert M., Yang W. (2020). Structure of an activated DNA-PK and its implications for NHEJ. Mol. Cell.

[40] Niewolik D., Schwarz K. (2022). Physical ARTEMIS:DNA-PKcs interaction is necessary for V(D)J recombination. Nucleic Acids Res..

[41] Shultz L.D., Ishikawa F., Greiner D.L. (2007). Humanized Mice in Translational Biomedical Research. Nat. Rev. Immunol..

[42] Kanaji N., Tadokoro A., Susaki K., Yokokura S., Ohmichi K., Haba R., Watanabe N., Bandoh S., Ishii T., Dobashi H. (2014). Higher Susceptibility of NOD/LtSz-scid Il2rg(−/−) NSG Mice to Xenotransplanted Lung Cancer Cell Lines. Cancer Manag. Res..

[43] Ito R., Takahashi T., Katano I., Ito M. (2012). Current Advances in Humanized Mouse Models. Cell. Mol. Immunol..

[44] Aryee K.E., Burzenski L.M., Yao L.C., Keck J.G., Greiner D.L., Shultz L.D., Brehm M.A. (2022). Enhanced Development of Functional Human NK cells in NOD-scid-IL2rg(null) Mice Expressing Human IL15. FASEB J..

[45] Ren J., Yu D., Fu R., An P., Sun R., Wang Z., Guo R., Li H., Zhang Y., Li Z. (2020). IL2RG-deficient Minipigs Generated via CRISPR/Cas9 Technology Support the Growth of Human Melanoma-derived Tumours. Cell Prolif..

